# Distinct and Intermediate Bacterial Community Structure of the Wasabi Rhizome Based on Compartment-resolved 16S rRNA Gene Profiling

**DOI:** 10.1264/jsme2.ME26005

**Published:** 2026-06-20

**Authors:** Masayoshi Hashimoto, Kana Ohata, Ryohei Thomas Nakano, Hisae Hirata, Yusuke Katai

**Affiliations:** 1 Faculty of Agriculture, Shizuoka University, Shizuoka, Japan; 2 Faculty of Science, Hokkaido University, Sapporo, Japan; 3 Izu Agricultural Research Center, Shizuoka Prefectural Research Institute of Agriculture and Forestry, Shizuoka, Japan

**Keywords:** Wasabi (*Eutrema japonicum*), 16S rRNA gene amplicon sequencing, plant microbiota, rhizome, diversity

## Abstract

The plant microbiota plays important roles in plant responses to biotic and abiotic stresses. Wasabi (*Eutrema japonicum*), a traditional crop indigenous to Japan, remains poorly characterized in terms of its associated bacterial community. In the present study, 16S rRNA gene amplicon sequencing revealed distinct bacterial community structures across plant and soil compartments. The rhizome microbiota, the edible plant organ used as a spice, exhibited intermediate characteristics between those of the root and leaf microbiota. These results provide fundamental insights into the wasabi-associated bacterial microbiota and identify the rhizome as a distinct microbial niche.

Plants closely interact with the surrounding microbial community, collectively referred to as the plant microbiota (Bulgarelli *et al.*, 2013). Over the past two decades, many studies have reported that the plant microbiota plays critical roles in plant responses to biotic and abiotic stresses (Harbort *et al.*, 2020; Nakano and Shimasaki, 2024; Du *et al.*, 2025). These findings strongly suggest the potential of engineering the functions of the plant microbiota for sustainable agricultural production (Copeland *et al.*, 2025). Therefore, characterizing the microbial community composition of crop species provides fundamental information for plant microbiota engineering.

Wasabi, or Japanese horseradish (*Eutrema japonicum*), is an indigenous crop in Japan that belongs to the family Brassicaceae and is well recognized as an indispensable ingredient in traditional Japanese cuisine (Yamane *et al.*, 2016). Its characteristic pungency is attributed to allyl isothiocyanate (AITC), an aliphatic glucosinolate (GLS)-derived secondary metabolite. The rhizomes, the main edible part of the wasabi plant, are morphologically classified as stem organs. In wasabi plants, the rhizome is located close to the soil surface, with its lower part giving rise to roots and being buried in the soil. This growth habit is also observed in other Brassicaceae species, such as *Raphanus sativus*. In addition, under traditional wasabi cultivation practices, wasabi rhizomes are continuously exposed to running water, typically at a water depth of approximately 1–2 cm. In many plant species, including the model plant *Arabidopsis thaliana*, soil is recognized as the primary microbial inoculum for belowground compartments, such as the roots and the rhizosphere (Bulgarelli *et al.*, 2012; Bai *et al.*, 2015; Zhu *et al.*, 2024; Fomekong *et al.*, 2025). Given the physical proximity of the rhizome to soil and continuous exposure to running water in wasabi cultivation, the rhizome may represent a distinct microbial niche that combines the characteristics of both above- and below-ground compartments. Moreover, the roots and the rhizosphere of wasabi may harbor distinct microbiomes due to continuous exposure to running water. However, to the best of our knowledge, no studies have characterized the rhizome-associated microbial community composition relative to those in other plant compartments in Brassicaceae species. In the present study, a 16S rRNA gene amplicon sequencing anal­ysis was performed to characterize the bacterial communities associated with the above- and below-ground parts of wasabi plants.

Mericlone seedlings of the wasabi cultivar Mazuma No. 1 (Miyoshi Agritech) were planted in April 2022 at a research field (34.89896° N, 138.87731° E; 340 m above sea level) located on the Izu Peninsula, Shizuoka Prefecture, Japan. The research field is managed by the Izu Agricultural Research Center, Shizuoka Prefectural Research Institute of Agriculture and Forestry. The research field was constructed using the traditional tatami-ishi-style cultivation system with continuous natural running water flushing throughout the experimental period (Fig. S1). Wasabi plants were grown in the field for six months prior to sample collection.

Four plant-derived compartments (the rhizome, root, leaf, and petiole; Fig. 1A) and three soil-derived compartments (bulk soil, the rhizosphere, and rhizome-associated soil compartment, hereafter referred to as the rhizomesphere) were harvested from each plant as described in Supplementary Methods. Soil chemical properties were measured by VEGETEC and are summarized in Table S1.

Amplicon sequencing was performed at the Bioengineering Lab using a two-step tailed PCR method targeting the V4 region of the bacterial 16S rRNA gene. Sequencing was performed on a MiSeq platform (Illumina) using MiSeq Reagent Kit v3 with 2×300 bp paired-end reads. Raw sequencing reads were deposited in the DDBJ DRA database under BioProject ID PRJDB42059. The reads were processed to obtain the feature table, taxonomy table, and representative sequences containing amplicon sequence variants (ASVs) according to Supplementary Methods.

A downstream anal­ysis, including data visualization and statistical tests, was performed in RStudio (v2025.05.1) (R Core Team, 2025), as described in Supplementary Methods. After removing non-bacterial ASVs (mitochondria, chloroplasts, and Archaea) from taxonomy and feature tables, 1,294,181 high-quality 16S rRNA reads were obtained from 35 samples. These samples comprised seven distinct compartments with five independent biological replicates per compartment. The average number of reads per sample was 36,977±10,673. These reads were rarefied to a sequencing depth of 21,178 sequences per sample.

To compare bacterial alpha diversity among compartments, we measured multiple alpha diversity indices for each sample (Fig. 1). The alpha diversity indices of the number of ASVs and the Shannon index were significantly higher in bulk and rhizosphere soils than in the other compartments (Fig. 1B and C). Consistent with previous studies on different plant species (Coleman-Derr *et al.*, 2016; Wagner *et al.*, 2016; Nakamura *et al.*, 2023), lower alpha diversity was observed in root and aboveground plant compartments than in bulk and rhizosphere soils. Alpha diversity was higher in the rhizomesphere than in the rhizome, but significantly lower than in bulk and rhizosphere soils, indicating that the rhizomesphere constitutes a distinct soil-associated microbial niche. Alpha diversity in the rhizome was similar to that in the root, although greater variations among biological replicates were observed in the rhizome than in the root. In terms of Pielou’s evenness index (Fig. 1D), which represents how balanced the distribution of species is in a microbial community, bulk and rhizosphere soils had significantly higher species evenness than plant compartments and the rhizomesphere. These results indicate that the plant compartments of wasabi plants and their associated soil compartments constitute distinct microbial niches with respect to alpha diversity and evenness.

To further compare microbial communities among plant and soil compartments, we performed a microbial source tracking anal­ysis across the compartments using the FEAST package (Shenhav *et al.*, 2019). Among soil compartments, bulk soil accounted for 93.9% of the rhizosphere community and 11.7% of the rhizomesphere community (Fig. 2A). In addition, 15.2% of the rhizomesphere community was derived from the rhizosphere. Regarding plant compartments, the root community was primarily derived from the rhizosphere (38.9%), whereas contributions from other soil compartments were less than 1% (0.81% from the rhizomesphere and 0.68% from bulk soil). In contrast, the rhizome community was mainly derived from the rhizomesphere (53.6%) and partially from the root (1.18%) and rhizosphere (0.86%). These results suggest that the root and rhizome microbiota were mainly attributed to soil-derived compartments, while the relative contributions of multiple soil-derived communities differed between the two compartments.

To characterize bacterial community structures across plant and soil compartments, we calculated Bray-Curtis dissimilarities between all pairs of samples. A principal coordinate anal­ysis (PCoA) showed that the first axis, explaining 26.35% of the variance, separated soil-derived compartments (bulk soil and rhizosphere), belowground plant compartments, and aboveground plant compartments (Fig. 2B). Samples of the rhizomesphere compartment clustered close to those of the rhizome compartment. Along the second axis, explaining 14.22% of the variance, root samples were separated from rhizome and rhizomesphere samples. A permutational multivariate anal­ysis of variance (PERMANOVA) consistently indicated that the compartment identity significantly explained the overall variation in‍ ‍wasabi-associated bacterial communities (*R^2^*=0.59, *P*<0.0001). In addition, all pairwise PERMANOVA comparisons between the compartments were significant.

To further evaluate the rhizome as a microbial niche, we compared between-compartment (with bulk soil as a common reference) and within-compartment Bray-Curtis distances (Fig. 2C and D). As shown in Fig. 2C, the two soil-derived compartments, the rhizosphere and rhizomesphere, were closer to bulk soil than the plant compartments, although their distances from bulk soil were significantly different from each other. Distances for root, rhizome, and leaf samples were significantly different and gradually increased in accordance with their physical separation from bulk soil. An anal­ysis of within-compartment dispersion revealed that root, rhizosphere, and bulk soil communities were the least dispersed, whereas rhizome and leaf communities were the most dispersed (Fig. 2D), suggesting that the rhizome community shares characteristics with the leaf community in terms of dispersion of the community structure. Higher dispersion in the rhizome and leaf communities may be attributed to greater heterogeneity in microbial compositions, as shown in Fig. 4 and S2, potentially resulting from lower microbial density, limited nutrient availability in aboveground tissue, and higher environmental fluctuations (Koskella, 2020; Sohrabi *et al.*, 2023). We also compared all pairs of plant compartments among the root, rhizome, and leaf (Fig. 2E). The largest distance occurred between root and leaf communities, while distances between the rhizome and either the root or leaf were similar. Collectively, these results indicate that the rhizome-associated bacterial community has intermediate characteristics between those of root and leaf compartments in terms of diversity metrics.

To compare the taxonomic composition of the wasabi-associated bacterial microbiota, we calculated the relative abundances of the detected taxa at the phylum level, with *Proteobacteria* being further resolved at the class level (Fig. 3). In total, 34 phyla were identified across all samples. The top 14 taxa are shown in Fig. 3. Across all belowground compartments, *Gammaproteobacteria* was the most abundant taxonomic group. In contrast, *Alphaproteobacteria* predominated in aboveground plant compartments. Among soil-derived compartments, *Alphaproteobacteria* was the second most abundant group in both bulk and rhizosphere soils, whereas *Bacteroidota* was the second most abundant only in the rhizomesphere. In the belowground plant compartments, *Bacteroidota* was the second most abundant group in both the root and rhizome. However, the third most abundant taxon differed between these compartments: *Actinobacteriota* in the root and *Alphaproteobacteria* in the rhizome. These results indicate that the wasabi-associated bacterial microbiota exhibits distinct higher-level taxonomic structures depending on plant and soil compartments.

We next investigated which bacterial taxa were enriched in each plant compartment at the family level. An anal­ysis of the top 50 bacterial families based on relative abundance revealed significant enrichment across plant compartments in 40 families (Fig. 4). Several families exhibited enrichment specific to individual plant compartments. For example, four‍ ‍families, *Rhodobacteraceae* (*Alphaproteobacteria*), *Rubritaleaceae*, *Verrucomicrobiaceae* (*Verrucomicrobiota*), and *Kaistiaceae* (*Alphaproteobacteria*), were significantly enriched in the rhizome. *Rhodobacteraceae* was highly abundant in the rhizome (8.0%). Although functional studies on *Rhodobacteraceae* in plants are limited, the present results appear to be consistent with previous studies reporting the dominance of *Rhodobacteraceae* in aquatic environments (Pohlner *et al.*, 2019; Isaac *et al.*, 2024; Roager *et al.*, 2024; Sun *et al.*, 2024), suggesting potential roles in the rhizome, possibly related to IAA production (Simon *et al.*, 2017). The *Rhodobacteraceae* strain, *Rhodobacter sphaeroides*, has been shown to exhibit bioremediation activity for cadmium (Cd)- and zinc (Zn)-contaminated soil (Peng *et al.*, 2018). *Rubritaleaceae* was previously identified as a responsive taxon under heavy metal-contaminated soil in the rhizosphere of *Miscanthus* x *giganteus* (Zadel *et al.*, 2020). *Rubritaleaceae* and *Kaistiaceae* have also been identified as enriched taxa in the tomato rhizosphere under low-nutrient hydroponic conditions (Mejia *et al.*, 2025). The enrichment of these taxa in the wasabi rhizome may be attributed to the traditional cultivation system, which involves continuous flushing with natural water and a stone-based structure.

Among the top 15 families, leaf-enriched taxa included *Sphingomonadaceae*, *Caulobacteraceae*, *Beijerinckiaceae* (*Alphaproteobacteria*), and *Sphingobacteriaceae* (*Bacteroidota*), whereas *Micromonosporaceae* (*Actinobacteriota*) was significantly enriched in the root. These taxa have frequently been detected in the corresponding compartments of various plant species and include strains with plant growth-promoting (PGP) activities, such as growth promotion, disease suppression, and IAA and siderophore production (Trujillo *et al.*, 2014; Vogel *et al.*, 2016; Krishnan *et al.*, 2017; Grossi *et al.*, 2020; Okazaki *et al.*, 2021), suggesting that these enriched taxa contribute to PGP functions in the leaf and root of wasabi.

In contrast, other families exhibited distinct abundance patterns, with some being commonly enriched across two plant compartments. Among the top 15 families, *Flavobacteriaceae*, *Chitinophagaceae*, and *Microscillaceae* (*Bacteroidota*) showed significantly higher abundance in both the rhizome and root than in the leaf, whereas *Rhizobiaceae* (*Alphaproteobacteria*) and *Spirosomaceae* (*Bacteroidota*) showed higher abundance in both the rhizome and leaf. These taxa have been reported to include strains with PGP activities in various plant species, such as growth promotion, heavy metal tolerance, disease suppression, phosphorus solubilization, and IAA and siderophore production (Soltani *et al.*, 2010; Walitang *et al.*, 2017; Vogel *et al.*, 2021; Muratova *et al.*, 2023; Srinivasan, 2024; Liu *et al.*, 2025). Therefore, the present results indicate that these taxa have the capacity to colonize the rhizome and exhibit PGP activities. However, no bacterial families were commonly enriched in both the root and leaf. Several families exhibited alternative enrichment patterns, including stepwise or partial distributions across the three plant compartments, such as *Comamonadaceae* (partial) and *Sphingomonadaceae* (stepwise). These taxon-level enrichment patterns may explain the intermediate characteristics of the rhizome-associated microbiota between those of the root and leaf.

At the genus level, plant compartment-dependent enrichment patterns were also observed in 39 of the top 46 bacterial genera (Fig. S2). Several genera exhibited significant enrichment specific to individual plant compartments. For example, three genera, unclassified *Comamonadaceae*, unclassified *Rhodobacteraceae*, and *Luteolibacter* (*Rubritaleaceae*), were specifically enriched in the rhizome compartment. Alternative enrichment patterns across the two plant compartments were also observed. Genera enriched across the rhizome and root included *Flavobacterium*, unclassified *Chitinophagaceae*, *Ferruginibacter*, *Microscillaceae*, *Tahibacter*, *Terrimonas*, and *env. OPS17*. Genera enriched across the rhizome and leaf included *Mycobacterium*, *Dyadobacter*, and *Nocardioides*. However, there were no commonly enriched genera in the root and leaf. In summary, these results indicate that the rhizome has a distinct microbial distribution pattern from other plant compartments, while partially sharing enriched taxa with the root and, to a lesser extent, with the leaf.

In the present study, we characterized bacterial communities associated with plant- and soil-derived compartments of wasabi using 16S rRNA gene amplicon sequencing. The rhizome, the main edible part of wasabi, is morphologically a stem-derived organ that enlarges in close proximity to the soil and represents a unique structure among Brassicaceae species (Yamashita *et al.*, 2024). Wassermann *et al.* (2017) previously compared bacterial communities among the edible tissues of several *Brassica* crops, including the roots of radish and horseradish, using commercially available samples. However, that study did not clearly distinguish the rhizome from the root or systematically characterize their microbiomes relative to those in other plant compartments. To the best of our knowledge, the present study provides the first comparative characterization of rhizome-associated bacterial communities across multiple plant compartments and surrounding soils in Brassicaceae plants.

Alpha diversity and source tracking anal­yses suggest a pattern reminiscent of the rhizosphere effect, in which the rhizome microbiota is selectively shaped from the surrounding environment, particularly the rhizomesphere (Bulgarelli *et al.*, 2012; Lundberg *et al.*, 2012; Lee *et al.*, 2019; Yamamoto *et al.*, 2020; Zhu *et al.*, 2024). A potential factor contributing to this selective microbial assembly process is the distinct chemical environment of the wasabi rhizome, including GLS-derived metabolites, such as AITC. AITC exhibits antimicrobial activity, making it suitable for soil fumigation and the prevention of foodborne illnesses associated with raw fish consumption. Zhu *et al.* (2020) showed that AITC-based soil fumigation significantly affected both bacterial and fungal community structures by enriching several microbial taxa, including *Sphingomonas* and *Streptomyces*. Importantly, GLSs comprise a highly diverse group of compounds that are mainly found in Brassicaceae plants. Their composition is known to markedly vary among plant species, developmental stages, and tissue types (Brown *et al.*, 2003). In addition, variations in allyl-GLS compositions were recently reported to significantly affect leaf-associated bacterial communities in natural populations of *A. thaliana* (Unger *et al.*, 2024) Therefore, these findings suggest the involvement of AITC in shaping the microbial community in wasabi. Consistent with this hypothesis, Di *et al.* (2022) demonstrated that sinigrin, a precursor of AITC, was the most abundant GLS in the rhizomes of two wasabi cultivars relative to those in‍ ‍other plant compartments. Accordingly, the abundance of‍ ‍the enriched taxa in the rhizome, including the *Rhodobacteraceae* family, may correlate with AITC accumulation levels. To further investigate the potential role of AITC in shaping the rhizome microbiota, it will be important to quantify AITC levels in each plant compartment of wasabi and to analyze which taxa correlate with the accumulation of AITC.

## Acknowledgements

We are grateful for the technical assistance of Ryoko Iwata and Hiroko Ohba at Shizuoka University. This research was supported by funds including JSPS KAKENHI (20K05955, 23K18023, 24K01892, and 25K22358), the Yamazaki Spice Promotion Foundation, Mishima Kaiun Memorial Foundation, and Kurita Water and Environment Foundation to M. H.

### Conflicts of interest

The authors declare that there are no conflicts of interest.

## Citation

Hashimoto, M., Ohata, K., Nakano, R. T., Hirata, H., and Katai, Y. (2026) Distinct and Intermediate Bacterial Community Structure of the Wasabi Rhizome Based on Compartment-resolved 16S rRNA Gene Profiling. *Microbes Environ ***41**: ME26005.

https://doi.org/10.1264/jsme2.ME26005

## Supplementary Material

Supplementary Material

## Figures and Tables

**Fig. 1.**
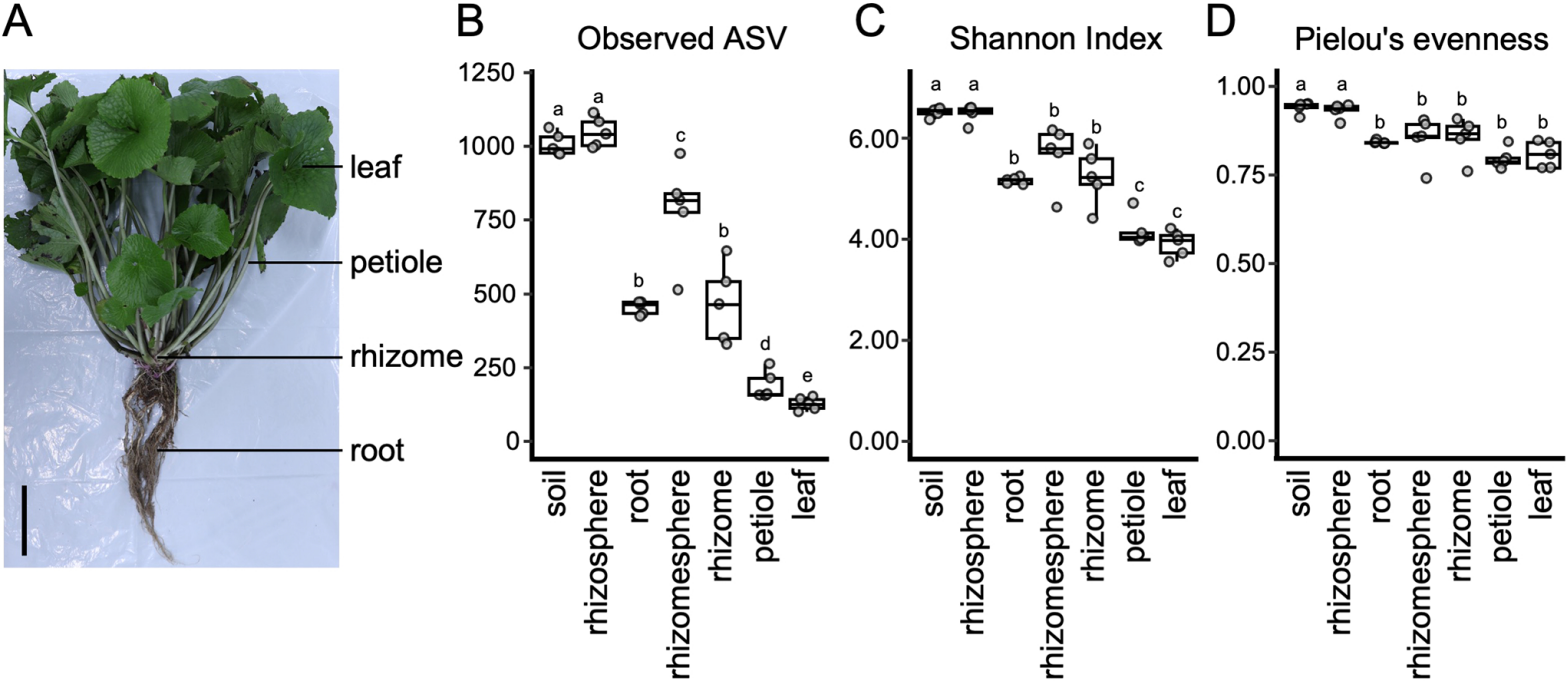
Alpha diversity of wasabi-associated bacterial communities across different compartments. (A) Representative wasabi plant cultivated for 6 months and harvested from the same field as the analyzed samples. Soil was gently washed with tap water. Scale bar=10 cm. (B, C, and D) Box plots showing the alpha diversity indices of (B) the observed number of amplicon sequence variants (ASVs), (C) the Shannon index, and (D) Pielou’s evenness. Each dot represents an individual sample. Boxes indicate the interquartile range (IQR; 25th–75th percentiles), with horizontal lines representing median values. Whiskers indicate minimum and maximum values excluding outliers. Outliers were defined as values exceeding 1.5×IQR above the upper quartile or below the lower quartile. Different letters within the same index indicate significant differences among compartments based on pairwise Wilcoxon rank-sum tests with the Benjamini–Hochberg adjustment (adjusted *P*<0.05).

**Fig. 2.**
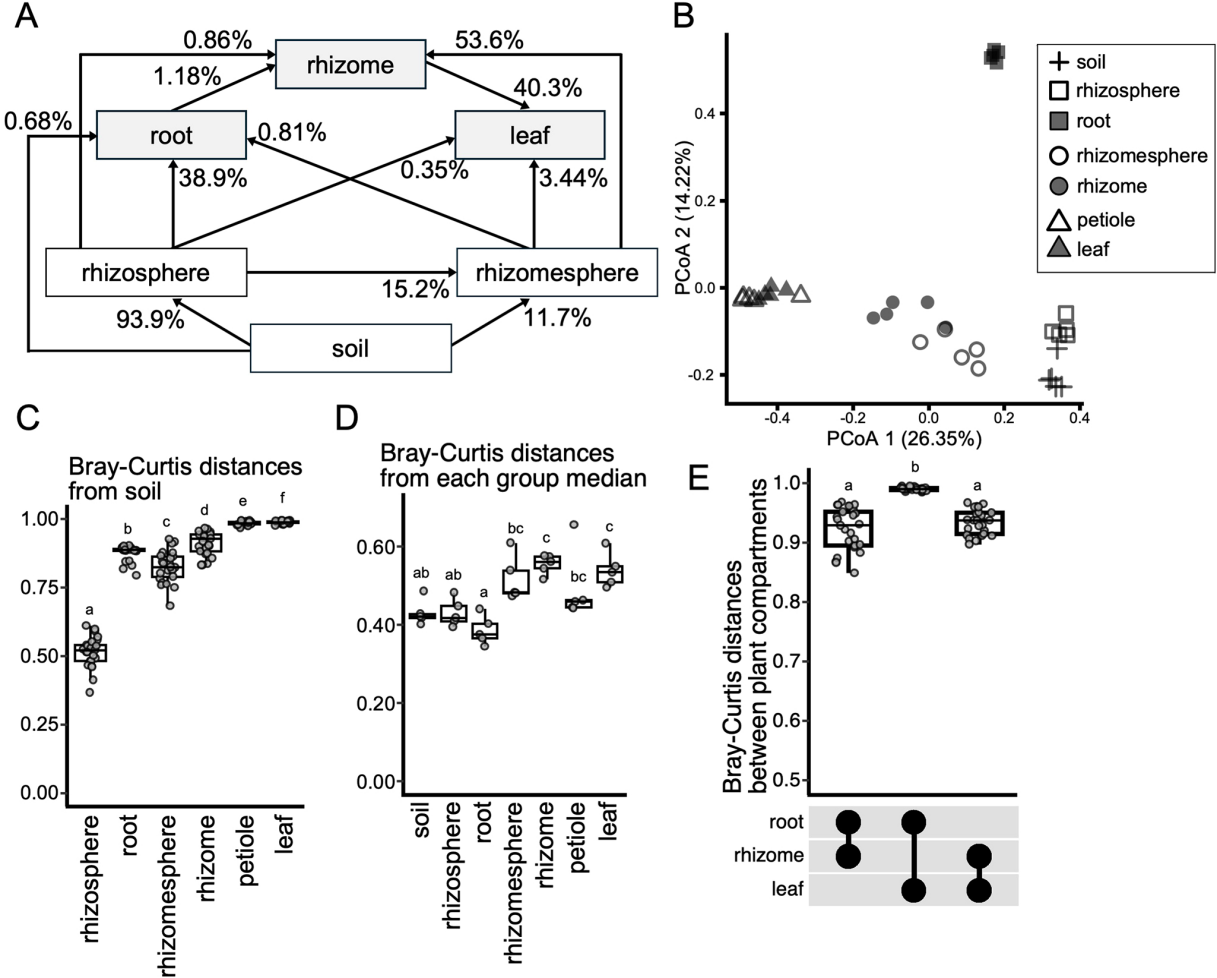
Comparisons of wasabi-associated bacterial communities across different compartments. (A) A microbial source tracking anal­ysis across compartments performed using the FEAST package. Plant and soil compartments were assigned as sinks and sources, respectively. Among soil compartments, bulk soil was assigned as the source relative to the rhizosphere, and bulk soil and the rhizosphere were assigned as sources relative to the rhizomesphere. Boxes corresponding to plant compartments are shown in gray. Sink–source relationships exceeding 0.001% are indicated by arrows. (B) A principal coordinate anal­ysis (PCoA) plot of beta diversity based on Bray–Curtis dissimilarities. Different shapes and colors represent different compartments. (C) Box plots of Bray–Curtis distances between each soil-compartment sample and samples from the other compartments. (D) Box plots of within-compartment dispersions calculated as Bray–Curtis distances from each sample to the compartment median. (E) Box plots of Bray–Curtis distances between every pair of samples from plant compartments (root, rhizome, and leaf). In the lower part of the plot, pairs of dots connected by lines indicate the plant compartment pairs being compared. Different letters in box plots indicate significant differences among conditions based on pairwise Wilcoxon rank-sum tests with the Benjamini–Hochberg adjustment (adjusted *P*<0.05).

**Fig. 4.**
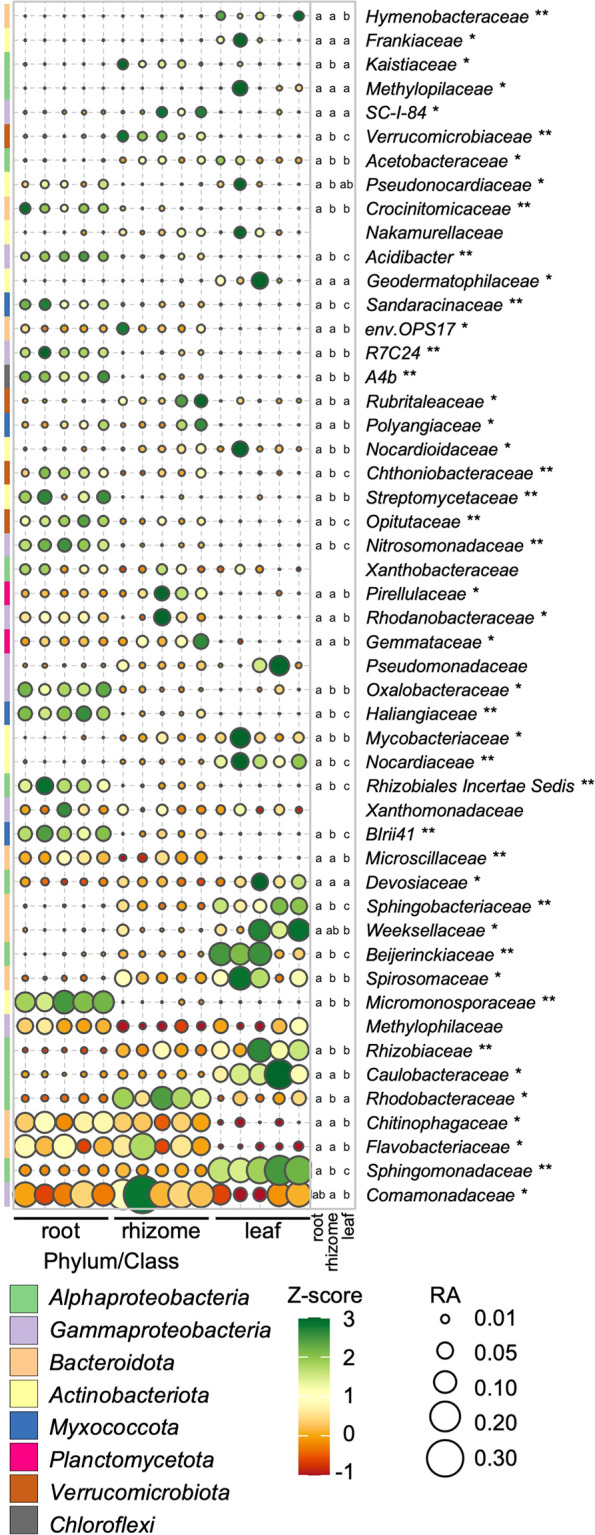
Distribution of dominant bacterial families across plant compartments. Bubble plots show the distribution of the top 50 bacterial families based on relative abundance (RA) across plant compartments. Each compartment consists of five biological replicates. Families are ordered according to the sum of their relative abundances across all samples. The size of each circle represents the RA of each family in each sample, and the color of each circle represents the Z-score calculated from RAs within each family across samples. The color code on the left indicates the phylum- or class-level taxonomic affiliation of each family. Asterisks indicate families with significant overall differences in RA among the three compartments based on the Kruskal-Wallis test with the Benjamini–Hochberg (BH) adjustment (**adjusted *P*<0.01, *adjusted *P*<0.05). Different letters on the right side of the plot indicate significant pairwise differences among compartments, based on post hoc Wilcoxon rank-sum tests with the BH adjustment (adjusted *P*<0.05).

**Fig. 3.**
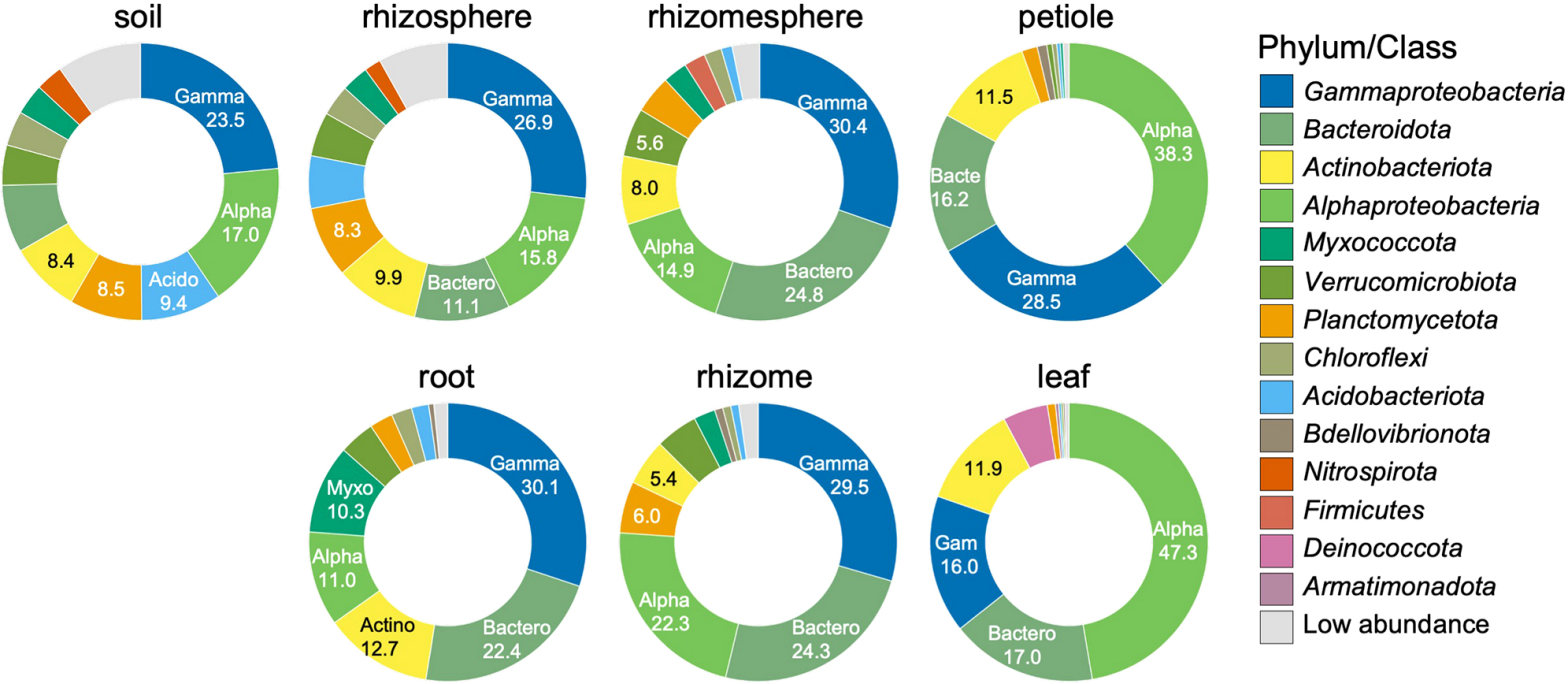
Composition of wasabi-associated bacterial communities across compartments. Pie charts show the average relative abundances of bacterial taxa in each compartment at the phylum level, except for *Proteobacteria*, which are shown at the class level.
