# Supplementary material for: Distinct and Intermediate Bacterial Community Structure of the Wasabi Rhizome Based on Compartment-resolved 16S rRNA Gene Profiling
Source: Microbes Environ. 2026 Jun 20;41(2):ME26005. doi: 10.1264/jsme2.ME26005 (PMC13293706; doi:10.1264/jsme2.ME26005)
Supplement: Supplementary file 1 — Supplementary Material [file 41_26005_s1.pdf]

Supplementary material

Distinct and intermediate bacterial community structure of the wasabi rhizome based on compartment-resolved 16S rRNA gene profiling

Masayoshi Hashimoto[1], Kana Ohata[1], Ryohei Thomas Nakano[2], Hisae Hirata[1], Yusuke Katai[3]
[1]Faculty of Agriculture, Shizuoka University, Shizuoka, JAPAN
[2]Faculty of Science, Hokkaido University, Sapporo, JAPAN
[3]Izu Agricultural Research Center, Shizuoka Prefectural Research Institute of Agriculture and Forestry, Shizuoka, JAPAN

Corresponding author: Masayoshi Hashimoto
TEL: +81-54-238-4824
E-mail: hashimoto.masayoshi@shizuoka.ac.jp

Supplementary Methods

Wasabi cultivation in the experimental field

The experimental field was managed by the Izu Agricultural Research Center, Shizuoka Prefectural Research Institute of Agriculture and Forestry, according to the common management practices described below. The field was constructed using the traditional tatami-ishi-style cultivation system and was continuously irrigated with natural water flowing across the field surface throughout the experimental period (Fig. S1). Irrigation water was supplied from a neighboring river and shared with the surrounding commercially managed fields. Water depth was maintained at approximately 1–2 cm by adjusting the inflow depending on the precipitation. On 25th April 2022, wasabi mericlone seedlings at the 4–5 expanded leaf stage (cultivar Mazuma No. 1; Miyoshi Agritech & Co., Ltd., Yamanashi, Japan) were planted at 25 cm × 20 cm intervals across the experimental field (8.6 m × 9.4 m). Each wasabi plant was individually surrounded by a polyvinyl chloride pipe (8.7 cm in diameter and 7.5 cm in height), which was half-buried into the field surface to prevent herbivory by aquatic insects. Weeds were manually removed as soon as they were observed. No fertilizer or agrochemicals were applied during cultivation. A shade net was used to prevent

high temperatures during summer. In commercial field, rhizomes are typically harvested approximately 1.5–2 years after transplantation. Wasabi plants were collected every six months to track microbiome dynamics during cultivation. The present study represents only the 6-month samples; additional time points will be presented in future work. On 31st October 2022, five individual wasabi plants without visible disease symptoms were randomly selected and were harvested together with their surrounding soil. Each plant was individually placed in a 45 L plastic bag, immediately transported to the laboratory at ambient temperature, and stored overnight in a cold room prior to sample processing.

Plant sample harvesting

Four plant-derived compartments (rhizome, root, leaf, and petiole) and three soil-derived compartments (bulk soil, rhizosphere, and rhizome-associated soil compartment, hereafter referred to as the rhizomesphere) were harvested from each individual plant according to the procedures described below. First, all aboveground tissues were separated from the belowground portion using sterile scissors. The belowground plant part with adhering soil was placed in a plastic bag (Unipack I-4, Seinsannipponsha Ltd., Tokyo, Japan) and was vigorously shaken by hand to remove the loosely attached soil. Approximately 500 mg of the detached soil was collected as the bulk soil compartment.

For petiole samples, six intact petioles were randomly selected from each plant. From each petiole, a 2-cm-long segment was excised from the middle region using a new disposable razor for each plant. The segments were washed with 5 mL of 70% ethanol containing 0.01% Tween 20 for 30 s by manual shaking in a 15-mL plastic tube. After washing, six 2-mm-long fragments were then excised from the central region of the washed petiole segments were collected as the petiole compartment. For leaf samples, five fully expanded leaves without visible disease symptoms or insect damage were randomly selected. From each leaf, a 1 × 2 cm leaf fragment was excised from the left side of the leaf. The leaf fragments were washed as described above for petiole samples. Subsequently, five fragments (2 mm thick and 1 cm in length) excised from the central region of the washed leaf fragments were collected as the leaf compartment. For root samples, roots were carefully separated from the rhizome using sterile scissors. Five roots were randomly selected, and a 5-cm-long root fragment was excised from the basal end of each root. The root fragments were washed twice for 30 s each with 15 mL PBS buffer (130 mM NaCl, 7 mM Na2HPO4·2H2O, 3 mM

$NaH_2PO_4 \cdot H_2O$, pH 7.0) by manual shaking in a 50-mL plastic tube. After washing, excess buffer was removed using PROWIPE Soft Wiper (Daio Paper Corporation, Tokyo, Japan). Five fragments (1 cm long) excised from the central region of the washed roots were collected as the root compartment. The remaining PBS buffer after root washing was combined into a 50-mL tube and centrifuged at 1,500 rpm for 20 min, and the resulting pellet was defined as the rhizosphere compartment. For rhizome samples, entire rhizomes were obtained by removing roots and petioles. The whole rhizome tissues were washed twice for 30s each with 20 mL PBS buffer in a plastic bag by manual rubbing (Unipack G-4). The remaining PBS buffer after rhizome washing was combined into a 50-mL plastic tube and centrifuged at 1,500 rpm for 20 min, and the resulting pellet was defined as the rhizomesphere (the soil compartment associated with rhizome). The washed rhizome was bisected longitudinally using a new disposable razor for each plant, and three fan-shaped tissue sections (2 mm thick) were excised horizontally from one half of each rhizome to represent the rhizome compartment. All collected samples were transferred into Lysing Matrix E tubes (MP Biomedicals, CA, USA) and stored at −80°C until DNA extraction.

Amplicon sequencing of the bacterial 16S rRNA gene

DNA extraction was performed using FastDNA SPIN Kit for Soil (MP Biomedicals) following the procedure with slight modifications to the homogenization step (Bai et al., 2015). For soil-derived samples, 978 µL Sodium Phosphate buffer and 122 µL of MT-buffer were added to each Lysing Matrix E tube, followed by two rounds of homogenization at 5.0 m/s for 30 s using a FastPrep 5G instrument (MP Biomedicals). For plant-derived samples, a 5-mm-diameter stainless steel bead (Bio Medical Science Inc., Tokyo, Japan) was placed into the matrix tube prior to the two rounds of sample homogenization. Then, Sodium Phosphate buffer and MT-buffer were added followed by additional two rounds of homogenization. DNA concentrations were quantified using Quant-iT PicoGreen dsDNA Assay Kit (Thermo Fisher Scientific, MA, USA) on a LightCycler 480 system (Roche, Basel, Switzerland). Amplicon sequencing was performed at the Bioengineering Lab (Kanagawa, Japan) using a two-step tailed PCR method. The V4 region of the bacterial 16S rRNA gene were amplified using primers, 515f_MIX (5′-ACACTCTTTCCCTACACGACGCTCTTCCGATCT-NNNNN-GTGCCAGCMGCCGCGGTAA-3′) and 806r_MIX (5′-

GTGACTGGAGTTCAGACGTGTGCTCTTCCGATCT-NNNNN-GGACTACHVGGGTWTCTAAT-3′) (Caporaso et al., 2011). To reduce amplification of plastid and mitochondrial DNA, the peptide nucleic acid (PNA) clamps, anti-plastid PNA (5′-GGCTCAACCCTGGACAG-3′) and anti-mitochondrial PNA (5′-GGCAAGTGTTCTTCGGA-3′), were included in the 1stPCR reaction (Lundberg et al., 2013). PCR products were purified using AMPure XP beads (BECKMAN COULTER, Inc., CA, USA) at a 1:0.7 volume ratio. Each PCR product from 1stPCR was individually dual-barcoded in 2ndPCR. Sequencing was performed on a MiSeq platform (Illumina, Inc., CA, USA) using MiSeq Reagent Kit v3 with 2 300 bp paired-end reads. The fastx_barcode_splitter tool from the FASTX-Toolkit (ver. 0.0.14) was used to demultiplex and extract the read sequences whose 5′-end perfectly matches the primer sequences. Primer sequences were removed using the fastx_trimer tool. Low-quality reads with less than 130 bp with quality scores <20 were discarded using the sickle tool (ver. 1.33). Paired-end reads were merged using FLASH (ver. 1.2.11). The DADA2 plugin in Qiime2 (ver. 2023.7) was used to discard chimeric reads, resulting in the representative sequences and the feature table containing amplicon sequence variants (ASVs) (Bolyen et al., 2019; Callahan et al., 2017, 2016). Taxonomy was assigned using feature-classifier plugin by comparing the representative sequences and SILVA (ver. 138) 99% OTUs database (Quast et al., 2013).

Statistical analysis of 16S amplicon sequencing

Downstream analysis including data visualization and statistical test was performed in RStudio (v2025.05.1) (R Core Team, 2025). After removing the non-bacterial ASVs (mitochondria, chloroplasts, and Archaea) from taxonomy and feature tables, reads were rarefied to a sequencing depth of 21,178 sequences per sample using rrarefy function in vegan package to retain all samples while minimizing sequencing depth bias. Alpha diversity indices (Observed ASVs, Shannon index, and Pielou’s evenness) were calculated using estimateR and diversity function in vegan package. Data was processed and visualized using reshape, ggforce, ggplot2 (v3.4.4), export, and rcompanion packages. Beta diversity was calculated and assessed using Principal Coordinates Analysis (PCoA) based on Bray-Curtis dissimilarities using vegdist and cmdscale functions in vegan package. Permutational multivariate analysis of variance (PERMANOVA) test was performed using adonis function in vegan package.

Microbial source tracking analysis was performed using FEAST package.

References

Bai, Y., Müller, D. B., Srinivas, G., Garrido-Oter, R., Potthoff, E., Rott, M., Dombrowski, N., Münch, P. C., Spaepen, S., Remus-Emsermann, M., Hüttel, B., McHardy, A. C., Vorholt, J. A., & Schulze-Lefert, P. (2015). Functional overlap of the Arabidopsis leaf and root microbiota. Nature, 528(7582), 364–369.

Bolyen, E., Rideout, J. R., Dillon, M. R., Bokulich, N. A., Abnet, C. C., Al-Ghalith, G. A., Alexander, H., Alm, E. J., Arumugam, M., Asnicar, F., Bai, Y., Bisanz, J. E., Bittinger, K., Brejnrod, A., Brislawn, C. J., Brown, C. T., Callahan, B. J., Caraballo-Rodríguez, A. M., Chase, J., … Caporaso, J. G. (2019). Reproducible, interactive, scalable and extensible microbiome data science using QIIME 2. Nature Biotechnology, 37(8), 852–857.

Callahan, B. J., McMurdie, P. J., & Holmes, S. P. (2017). Exact sequence variants should replace operational taxonomic units in marker-gene data analysis. The ISME Journal, 11(12), 2639–2643.

Callahan, B. J., McMurdie, P. J., Rosen, M. J., Han, A. W., Johnson, A. J. A., & Holmes, S. P. (2016). DADA2: High-resolution sample inference from Illumina amplicon data. Nature Methods, 13(7), 581–583.

Caporaso, J. G., Lauber, C. L., Walters, W. A., Berg-Lyons, D., Lozupone, C. A., Turnbaugh, P. J., Fierer, N., & Knight, R. (2011). Global patterns of 16S rRNA diversity at a depth of millions of sequences per sample. Proceedings of the National Academy of Sciences of the United States of America, 108 Suppl 1(supplement_1), 4516–4522.

Lundberg, D. S., Yourstone, S., Mieczkowski, P., Jones, C. D., & Dangl, J. L. (2013). Practical innovations for high-throughput amplicon sequencing. Nature Methods, 10(10), 999–1002.

Quast, C., Pruesse, E., Yilmaz, P., Gerken, J., Schweer, T., Yarza, P., Peplies, J., & Glöckner, F. O. (2013). The SILVA ribosomal RNA gene database project: improved data processing and web-based tools. Nucleic Acids Research, 41(Database issue), D590-6.

R Core Team. (2025). R: A language and environment for statistical computing. R Foundation for Statistical Computing.

Supplemental Figures

Fig. S1. Tatami-ishi-style wasabi cultivation system.

(A) Cartoon illustration of a cross section of the tatami-ishi-style system used for traditional wasabi cultivation. Wasabi fields are constructed in a stair-step structure using natural stones. Water that overflows from the upstream canal flows across the surface of the field toward the downstream canal. The water then reaches the upstream side of the next lower step through an irrigation canal. Large stones form the bottom layer, medium-sized stones form the middle layer, and small stones and sand form the surface layer. This structure allows surface running water to flow vertically through the spaces between the stones. As a result, the water from the upper step flows down to the next lower step. Wasabi plants are cultivated on the flat surface of each step. Blue arrows indicate the direction of water flow.

(B) Photograph of the experimental field used in this study. Photographs were taken at March 2026. The left photo was taken looking upstream from downstream, and the right photo was taken from the upper step toward the field.

Fig. S2. Distribution of dominant bacterial genera across plant compartments.

Bubble plots show the distribution of the top 46 bacterial genera based on relative abundance (RA) across plant compartments. Each compartment consists of five biological replicates. Amplicon sequence variants (ASVs) that could not be classified at the genus level were grouped as “unclassified genus” within their respective families. Genera are ordered according to the sum of their relative abundances across all samples. The size of each circle represents RA of each genus in each sample, and the color of each circle represents the Z-score calculated from RAs within each genus across samples. The color code on the left indicates the phylum- or class-level taxonomic affiliation of each genus. Asterisks indicate genera with significant overall differences in RA among the three compartments based on the Kruskal-Wallis test with Benjamini–Hochberg (BH) adjustment (**adjusted $P < 0.01$, *adjusted $P < 0.05$). Different letters on the right side of the plot indicate statistically significant pairwise differences among compartments, based on post hoc Wilcoxon rank-sum tests with BH adjustment (adjusted $P < 0.05$).

Supplemental Tables

Table S1

Chemical properties of the tested wasabi field soil

item	value	unit
pH (H_2O)	7.3	
EC	0.04	mS/cm
Exchangeable calcium (CaO)	239	mg/100g
Exchangeable magnesium (MgO)	45.7	mg/100g
Exchangeable potassium (K_2O)	30.0	mg/100g
Cation exchange capacity (CEC)	12.9	meq/100g
Base saturation percentage	88.8	%
Available phosphorus (P_2O_5)	<3.0	mg/100g
Ammonium nitrate (NH_4-N)	0.72	mg/100g
Nitrate (NO_3-N)	0.58	mg/100g
phosphate absorption coefficient	542	mg/100g
humus	<0.9	%
available iron (Fe)	16.3	mg/kg
available manganese (Mg)	12.8	mg/kg
available zinc (Zn)	3.04	mg/kg
available copper (Cu)	0.51	mg/kg

Fig. S1

A

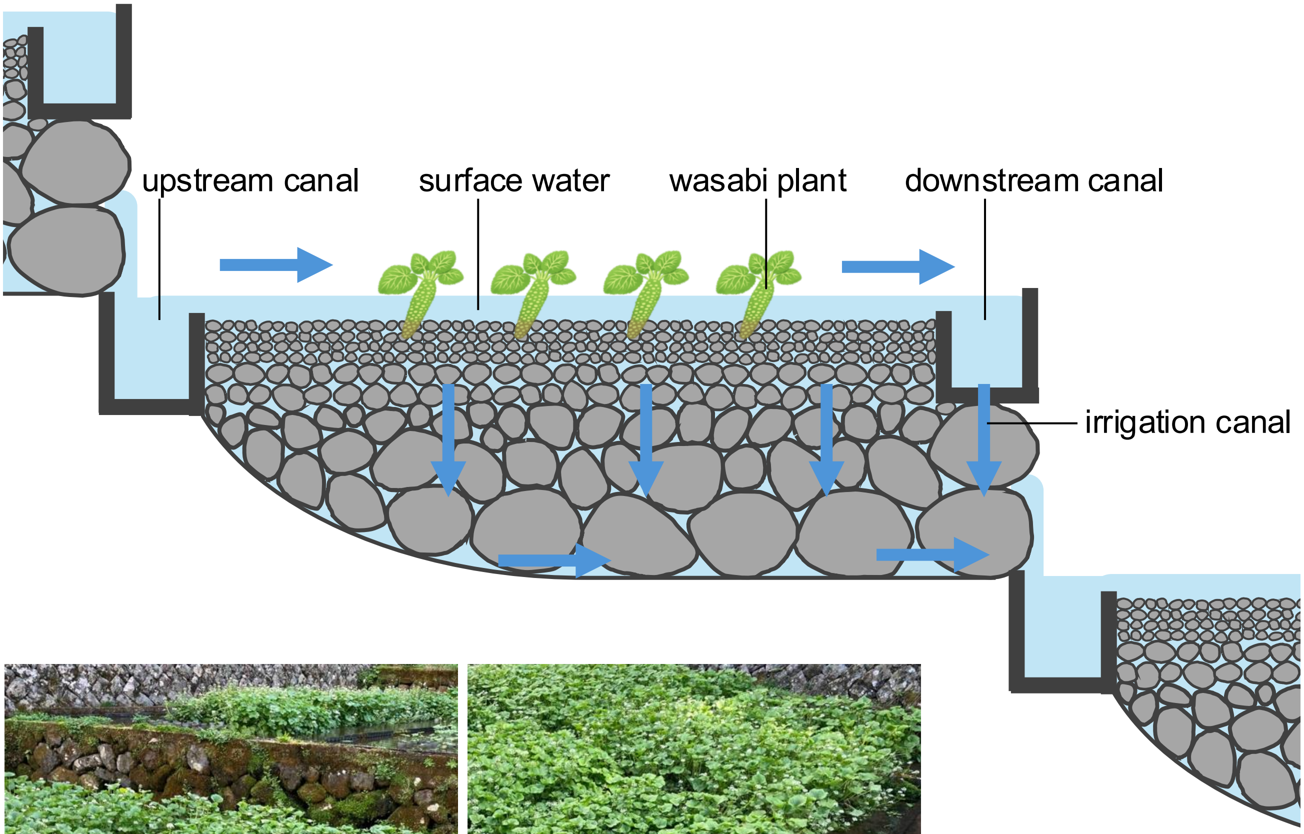

B

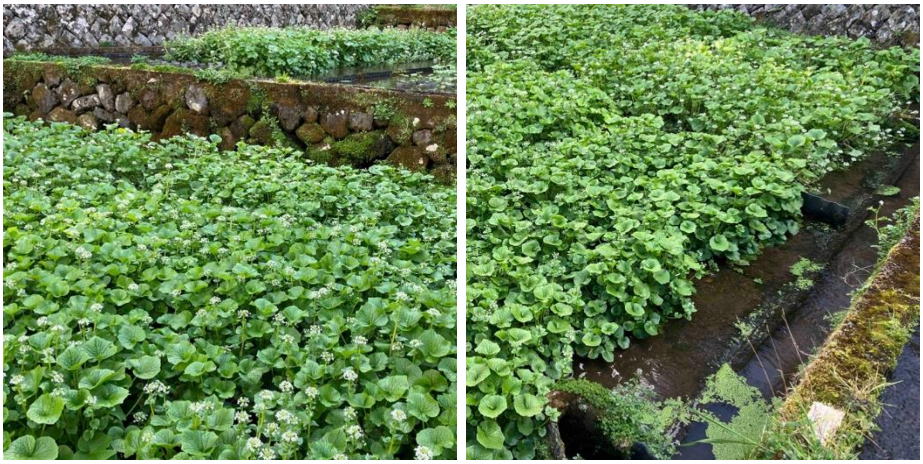

Figure S1. Tatami-ishi-style wasabi cultivation system.
(A) Cartoon illustration of a cross section of the tatami-ishi-style system used for traditional wasabi cultivation. Wasabi fields are constructed in a stair-step structure using natural stones. Water that overflows from the upstream canal flows across the surface of the field toward the downstream canal. The water then reaches the upstream side of the next lower step through an irrigation canal. Large stones form the bottom layer, medium-sized stones form the middle layer, and small stones and sand form the surface layer. This structure allows surface running water to flow vertically through the spaces between the stones. As a result, the water from the upper step flows down to the next lower step. Wasabi plants are cultivated on the flat surface of each step. Blue arrows indicate the direction of water flow.
(B) Photograph of the experimental field used in this study. Photographs were taken at March 2026. The left photo was taken looking upstream from downstream, and the right photo was taken from the upper step toward the field.

Fig. S2

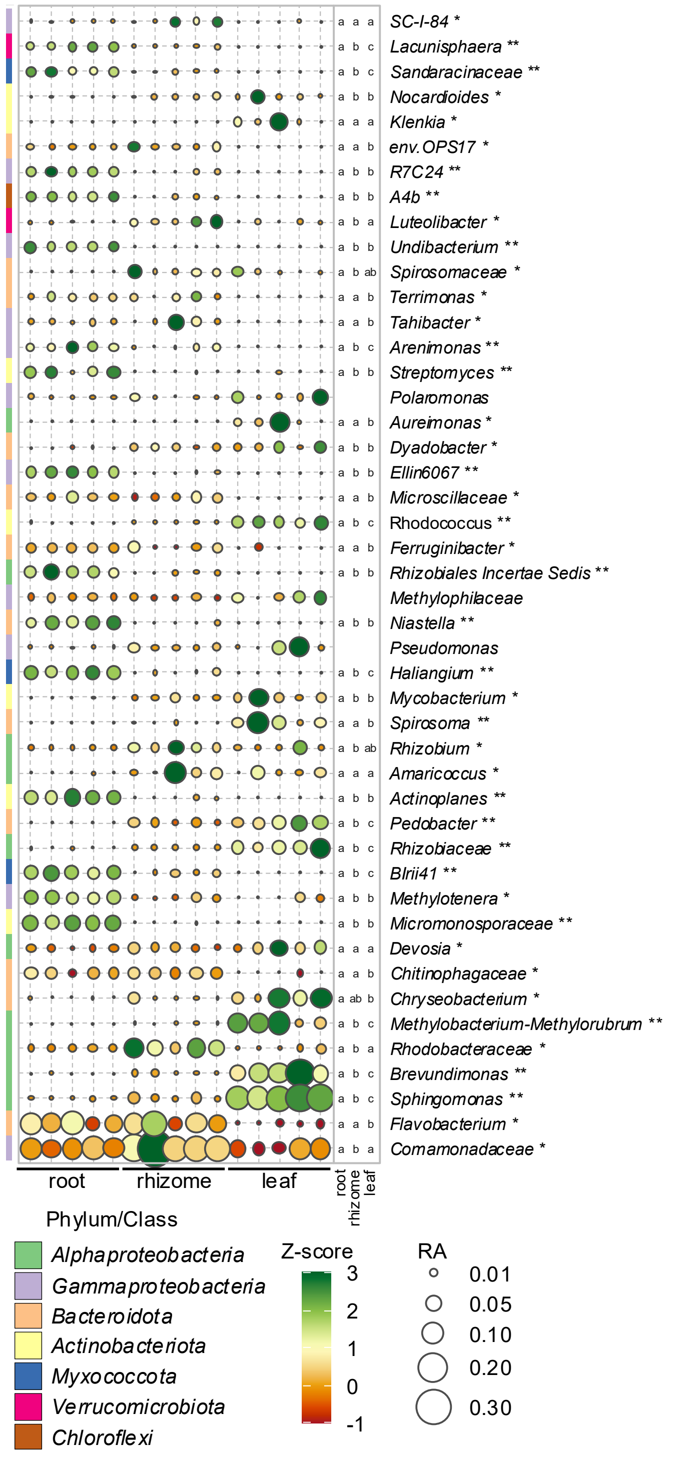

Figure S2. Distribution of dominant bacterial genera across plant compartments. Bubble plots show the distribution of the top 46 bacterial genera based on relative abundance (RA) across plant compartments. Each compartment consists of five biological replicates. Amplicon sequence variants (ASVs) that could not be classified at the genus level were grouped as “unclassified genus” within their respective families. Genera are ordered according to the sum of their relative abundances across all samples. The size of each circle represents RA of each genus in each sample, and the color of each circle represents the Z-score calculated from RAs within each genus across samples. The color code on the left indicates the phylum- or class-level taxonomic affiliation of each genus. Asterisks indicate genera with significant overall differences in RA among the three compartments based on the Kruskal-Wallis test with Benjamini–Hochberg (BH) adjustment (**adjusted *P* < 0.01, *adjusted *P* < 0.05). Different letters on the right side of the plot indicate statistically significant pairwise differences among compartments, based on post hoc Wilcoxon rank-sum tests with BH adjustment (adjusted *P* < 0.05).
